# Developing the Current General Practitioner Programme to Improve Effectiveness

**DOI:** 10.1192/bjo.2026.11368

**Published:** 2026-06-30

**Authors:** Akshita Dandawate

**Affiliations:** Mersey Care NHS Trust, Liverpool, United Kingdom

## Abstract

**Aims:**

Previous project conducted within the trust hypothesised and found that a tailored General Practitioner (GP) teaching programme was preferred by trainees and met GP training needs when given in addition to the general teaching programmethat is available to all psychiatric and GP trainees. The recommendation at the time was to develop a wide range of topics within this programme that were aligned with the GP curriculum for psychiatry.

The aims of this project were to evaluate if the current programme is useful and relevant to GP trainees as well as aligned with GP curriculum. To make amendments to the programme to ensure this and therefore, improve the overall quality.

**Methods:**

I conducted two cycles with two GP trainee cohorts. In cycle 1: edited PowerPoints from previously designed GP programme with pre-chosen topics, delivered 6 teaching sessions and collected post session feedback on relevance, usefulness as well as qualitative data on what went well and what could be improved.

In cycle 2: Made changes to topics from previous cycle feedback and mapped topics against GP curriculum, formalised a timetable and information letter which was disseminated for smoother process, delivered 6 teaching sessions, modified feedback forms to collect both pre session feedback on confidence in topic and expectations and post session feedback on confidence in topic, expectations, relevance, usefulness and qualitative data on what went well and what could be improved.

**Results:**

The results in cycle 1 showed 88% found the teaching on risk assessment useful and100% found it relevant. However, it reached 100% positive response for usefulness and relevance by the end of cycle 2. Furthermore, the teaching on Balint groups was rated relevant by 50% of trainees and 0% found it useful. Therefore, it was replaced with teaching on personality disorders, which reached 100% positive response for usefulness and relevance in cycle 2.

Overall, in cycle 2, confidence rating increased after the teaching session for all trainees. In the qualitative feedback, overall satisfaction was high and some minor adjustments were asked for, mostly to include more sessions with more sub-speciality specific teaching.

**Conclusion:**

Possible direction for the future would be to take the recommendations from qualitative feedback forward into the next cycle. All topics to continue into the next cycle with additional topics, if possible on subspecialities. Another would be to continue mapping learning objectives to GP curriculum to maintain relevance.

